# Cisplatin-enriching cancer stem cells confer multidrug resistance in non-small cell lung cancer via enhancing TRIB1/HDAC activity

**DOI:** 10.1038/cddis.2016.409

**Published:** 2017-04-13

**Authors:** Lihui Wang, Xing Liu, Yong Ren, Jingyuan Zhang, Junli Chen, Wenlong Zhou, Wei Guo, Xiaoxuan Wang, Huiping Chen, Meng Li, Xiangzhong Yuan, Xun Zhang, Jingyu Yang, Chunfu Wu

**Affiliations:** 1 Department of Pharmacology, Shenyang Pharmaceutical University College of Life Science and Biopharmaceutical, Shenyang, China; 2 Benxi Institute of Pharmaceutical Research, Shenyang Pharmaceutical University, Shenyang, China; 3 Department of Pathology, Wuhan General Hospital of Guangzhou Command, People's Liberation Army, Wuhan, China

## Abstract

Chemotherapeutic agents are generally used as a frontline therapy for non-small cell lung cancer (NSCLC). However, resistance to chemotherapy arises rapidly in NSCLC, and the reasons for chemotherapy resistance have not been fully determined. Here, we found cisplatin, but not paclitaxel and doxorubicin, induced the enrichment of cancer stem cell (CSC) and conferred multidrug resistance in NSCLC cell lines. *In vivo* study confirmed drug-resistant tumors displayed the enhanced expressions of CSC transcription factors. Mechanistically, cisplatin treatment resulted in C/EBP-*β*-dependent increasing of TRIB1. The crucial role of TRIB1 in cisplatin-induced enrichment of CSC and drug resistance was verified by knockdown TRIB1. Interestingly, cisplatin treatment also contributed to the increasement of HDAC, the interaction of TRIB1 with HDAC, and inactivation of p53. Similarly, the silencing of HDAC led to reduction of cisplatin-induced CSC, and combined knockdown of HDAC and TRIB1 exhibited enhanced effect. Additionally, the combination of HDAC inhibitor and cisplatin showed a reinforced antitumor action in NSCLC cell lines with TRIB1-dependent manner and remarkably shrink tumors in xenograft models. Moreover, cisplatin-treated NSCLC patients with high levels of TRIB1 exhibited a significantly poorer prognosis. Our findings illustrate a novel perspective in the evolution of chemotherapy resistance and provide a promising approach for the treatment of patients with NSCLC.

## 

Non-small cell lung cancer (NSCLC) accounts for ~80% of all lung cancers,^1^ and chemotherapy is the standard therapeutic approach to treat patients with advanced NSCLC.^2^ Although chemotherapy is initially effective, the patients inevitably encounter the chemotherapy resistance, subsequently are at a high risk of local regional recurrence and distant relapse.^3, 4^ Therefore, discovery of novel resistance mechanisms and based strategies to suppress chemotherapy resistance are especially helpful for the management of advanced NSCLC.

The cancer stem cells (CSCs) theory postulates that a subset of cancer cells share characteristics of normal stem cells, with a capacity of self-renewal and differentiation.^5^ Growing evidence have demonstrated that CSCs could protect against chemotherapeutic agents by means of various mechanism, such as induction metabolic enzyme aldehyde dehydrogenase (ALDH),^6^ overexpression of ABC transporters,^7^ activation of Notch, Hedgehog and Wnt pathways.^8^ Furthermore, the CSCs own elevated tumor-initiating capacity and metastasis-forming potential,^9, 10^ which display overlapping phenotypes with patients of acquired chemotherapy resistance, such as local regional recurrence and distant relapse. Given the multiplicity of genetic and environmental stimuli that are the origin of CSCs,^11^ it is equally not surprising that chemotherapy agents might be involved in the induction and maintenance of CSCs.

The Trib family of proteins encodes serine/threonine kinase like proteins, also called pseudokinases, which act as adaptor proteins in several signaling pathways.^12^ Among the Trib members, TRIB1 has been identified as oncogene that mediates oncogenesis through the MEK–ERK pathway, GRP78–Akt pathway and C/EBP regulation.^13, 14^ TRIB1 is known to be overexpressed in leukemia and prostate cancer, and its activation may promote aggressive growth and apoptotic resistance.^12, 13, 14^ Recently, Tang *et al.*^15^ reported that Trib1 also played a critical role in the development of radioresistance of glioma cells, suggesting the multiple roles of TRIB1 in the progression and therapy of cancer.

In this study, we show that cisplatin pretreatment significantly enriched CSCs of NSCLC cells *in vitro* and *in vivo*, and this enrichment was mediated by activation of TRIB1 and HDAC complex. In addition, the combination of HDAC inhibitor and cisplatin exhibited a reinforced antitumor action in NSCLC cell lines and xenograft models. Moreover, the present results suggest that TRIB1 may be a valuable predictive biomarker for NSCLC's patients with cisplatin treatment.

## Results

### Chemotherapy agents pretreatment displayed various action on CSCs in NSCLC

**Figure 1:**
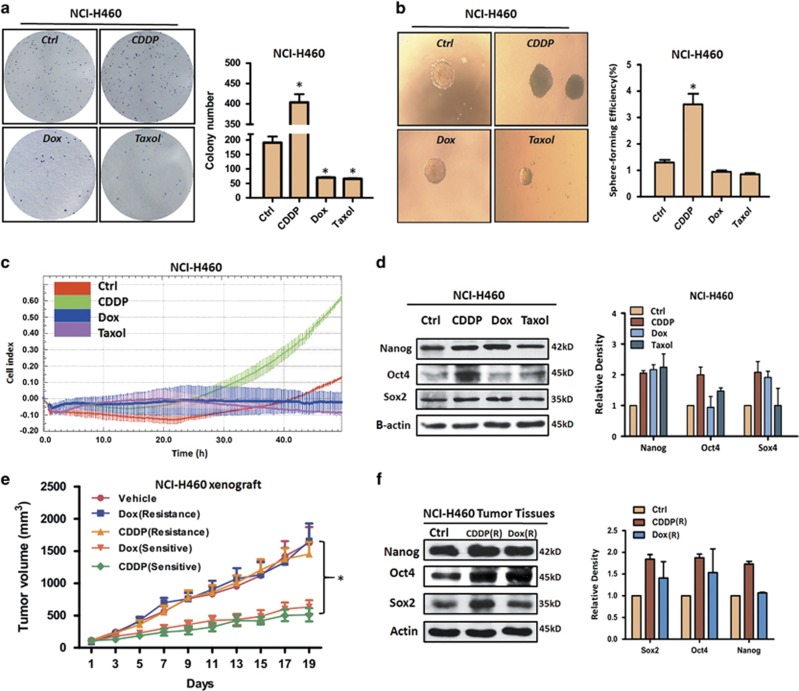
The effects of chemotherapy agents on CSCs characteristics in NSCLC cells. (**a**) The colony formation of NCI-H460 cells after pretreatment with CDDP (10 *μ*M), Dox (0.25 *μ*M) and Taxol (0.125 *μ*M). (**b**) The sphere formation of NCI-H460 cells after pretreatment with CDDP (10 *μ*M), Dox (0.25 *μ*M) and Taxol (0.125 *μ*M). (**c**) The migration of NCI-H460 cells after pretreatment with chemotherapy agents as above described concentration. The cell index represents the capacity for cell migration, and the slope of the curve can be related to the migration velocity of tumor cells. (**d**) The effects of pretreatment with chemotherapy agents on the expression of Nanog, Oct4 and Sox2 in NCI-H460 cells. *β*-Actin expression was used as a loading control. (**e**) The tumor growth status after administrated with Dox (2.2 mg/kg/3–4days, i.v.; *n*=6) or CDDP (5.5 mg/kg/3–4days, i.v.; *n*=6) for 19 days. (**f**) The expression levels of Nanog, Oct4 and Sox2 in vehicle control tumors, CDDP resistant tumors and Dox resistant tumors. *β*-Actin expression was used as a loading control. All error bars are s.e.m. *P<0.05, compared with control

The colony-forming assay is used to study the proliferation and differentiation pattern of cancer stem cell by their ability to form colonies. Our results showed that, under pharmacological concentrations, cisplatin (CDDP) pretreatment could result in significant increase of colony formation of NCI-H460 (p53 wild-type genotype) cells as compared with DMSO control, whereas addition of doxorubicin (Dox) and paclitaxel (Taxol) to NCI-H460 cells led to a decrease of colony number (Figure 1a). Similar pattern was also exhibited in A549 (p53 wild-type genotype) and NCI-H1299 (p53-null genotype cells) (Supplementary Figure 1A). Next, we measured the effects of chemotherapy agents on sphere formation, which reflects self-renewal capability. As shown in Figure 1b, CDDP pretreatment, but not Dox and Taxol, contributed to the enhancement of sphere formation ability of NCI-H460 cells. The data was well confirmed by NCI-H1299 cells (Supplementary Figure 1B). The migratory ability is considered as an important characteristics of CSCs,^16^ thus we also detected the changes of cell migration after treated with chemotherapy agents. Real-time cell analysis data indicated that only treatment with CDDP promoted cell migration as compared with DMSO control (Figure 1c). The above phenotypic results preliminarily demonstrate that CDDP, but not Dox and Taxol, enriches CSCs in NSCLC independent of p53 status.

Pluripotency transcription factors, including Nanog, Oct4 and Sox2, have a crucial role in initiating and maintaining CSCs,^17^ so we assessed the effect of chemotherapy agents on the expression level of them in NSCLCs. As shown in Figure 1d, the expression level of Nanog in NCI-H460 cells was upregulated by three chemotherapy agents, whereas, for Oct4 and Sox2, three chemotherapy agents exhibited different regulation. Among them, CDDP showed unique upregulation on Nanog, Oct4 and Sox2, which is consistent with our phenotypic data. Similar expression pattern was also showed in A549 cells (Supplementary Figure 1C). Subsequently, we further confirmed the above data using *in vivo* model. After administrated with CDDP and Dox for 19 days, the NCI-H460 xenograft mice models displayed various sensitivity (Figure 1e). About half of mice showed a resistant to CDDP and Dox treatment. Importantly, western blot results indicated, compared with vehicle control group, pluripotency transcription factors, including Nanog, Oct4 and Sox2, were upregulated in CDDP resistant tumors (Figure 1f). In addition, the expression levels of Nanog and Oct4 were also increased in Dox resistant tumors. Taken together, these results illustrate that CDDP induces the enrichment of CSCs in NSCLC, which might be associated with chemotherapy resistance.

### Cisplatin pretreatment induced CSCs markers and conferred multi-resistance in NSCLC

**Figure 2:**
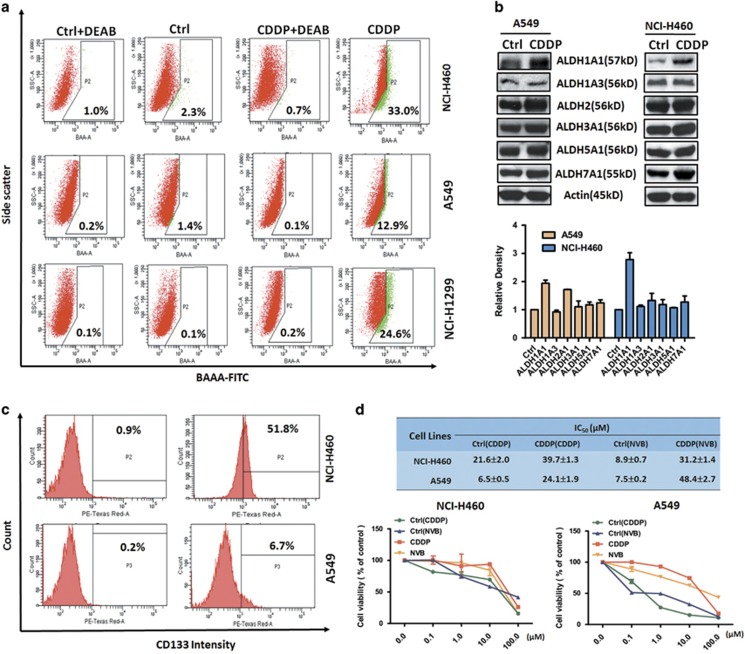
The effects of cisplatin pretreatment on CSCs markers and multi-resistance in NSCLC. (**a**) ALDH activity was detected by flow cytometry in CDDP-pretreated and DMSO-pretreated NCI-H460, A549 and NCI-H1299 cells. DEAB was used as ALDH inhibitor. (**b**) The ALDH subtypes, including ALDH1A1, ADLH1A3, ALDH2, ALDH3A1, ALDH5A1 and ALDH7A1 were measured in CDDP-pretreated and control NCI-H460 cells. *β*-actin expression was used as a loading control. (**c**) The CD133 expression was detected by flow cytometry in CDDP-pretreated and control NCI-H460 and A549 cells. (**d**) The cell viability of CDDP-pretreated or control NCI-H460 and A549 cells after treated with different concentrations of CDDP and NVB for 48 h

ALDH and CD133 are considered as classical and reliable biomarkers of CSCs in NSCLC.^18^ To further substantiate the results found in CDDP-treated NSCLC cells, we detected the effects of pretreatment with CDDP on ALDH and CD133 in NSCLC cells. The data from flow cytometry study is presented in Figure 2a, CDDP pretreatment could enhance the ALDH activity in NCI-H460 (from 0.7 to 33.0%), A549 (from 0.1 to 12.9%) and NCI-H1299 (from 0.2 to 24.6%) cells. Consistent with the flow cytometry data, western blot result showed CDDP pretreatment resulted in an upregulation of ALDH subtypes (Figure 2b), especially ALDH1A1, which predominantly attributes to ALDH activity.^19^ Moreover, we also found that CD133 expression was increased in NCI-H460 cells and A549 cells (Figure 2c), but not NCI-H1299 cells (data not shown), after CDDP pretreatment. The above results indicate CDDP pretreatment can induce CSCs markers in NSCLC cells which further confirmed the action of CDDP on CSCs enrichment.

Drug resistance is major contributors to chemotherapy failure, and also a crucial characteristics of CSCs.^18^ To address whether CDDP-pretreated and untreated NSCLC cells differ in drug sensitivity, the cells was exposure to CDDP itself and vinorelbine (NVB), a first line drug for NSCLC. Our results revealed that pretreated with CDDP contributed to a resistance to both CDDP and NVB, with 1.8–3.5 folds increase of IC_50_ values for CDDP and 3.7–6.5 folds increase of IC_50_ values for NVB (Figure 2d), which is consistent with our *in vivo* data (Figure 1e), suggesting CDDP treatment could mediate multi-resistance in NSCLC.

### Upregulation of TRIB1 by C/EBP-*β*-mediated CDDP induced enrichment of CSCs in NSCLC cells

**Figure 3:**
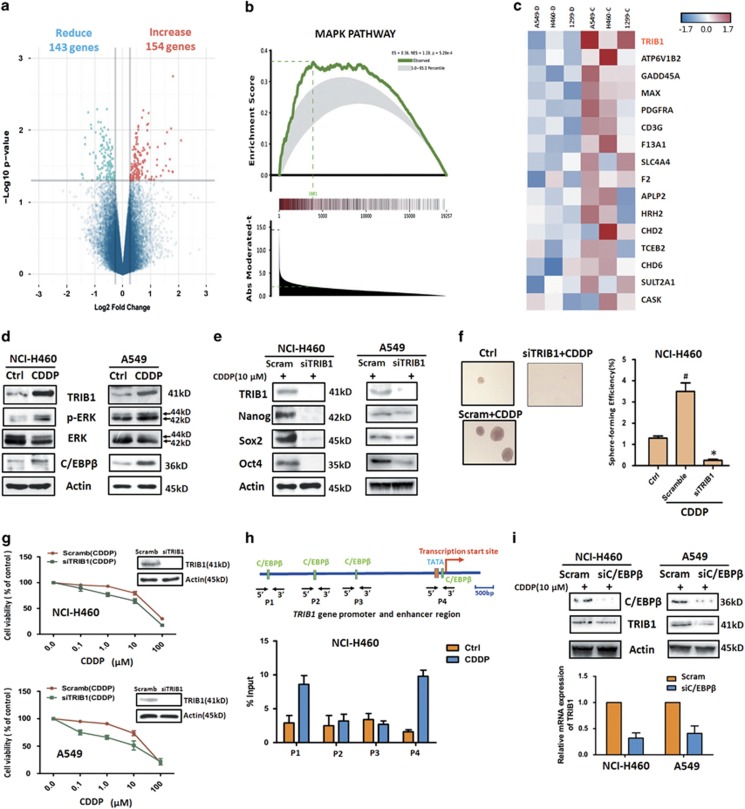
Microarray gene expression analysis and TRIB1 role in CDDP induced enrichment of CSCs. (**a**) Volcano plot showing fold changes and *P-*values for a particular comparison (CDDP-pretreated versus control cells in this case) in A549, NCI-H460 and NCI-H1299 cells. (**b**) GSEA of microarray data sets reveals enrichment of MAPK pathway in CDDP-pretreated three NSCLC cell lines. ES, enrichment score; NES, normal enrichment score. (**c**) Heat map depicting the differential expression of MAPK related genes between CDDP-pretreated and control three NSCLC cell lines. Pink and blue indicate high and low mRNA expression levels, respectively. (**d**) The expression levels of TRIB1, phosphor-ERK, ERK and C/EBP-*β* were detected by western blot in CDDP-pretreated and control NCI-H460 and A549 cells. *β*-Actin expression was used as a loading control. The expression CSCs transcription factors (**e**), sphere formation (**f**) and sensitivity to CDDP (**g**) were accessed in CDDP-pretreated NCI-H460 or/and A549 cells after knockdown TRIB1 by siRNA (33 nM). (**h**) ChIP assays confirmed the binding of C/EBP-*β* to the promoter and enhancer regions upstream of TRIB1 gene. (**i**) The expressions of C/EBP-*β* and TRIB1 were assessed by western blot or real-time RT-PCR in CDDP-pretreated NCI-H460 and A549 cells after knockdown C/EBP-*β* by siRNA (50 nM). All error bars are s.e.m. ^**#**^*P*<0.05, compare with control; *P<0.05, compare with Scramble

To identify the mechanisms underlying the observed CSCs enrichment and resistance induced by CDDP, microarray gene expression analysis was performed using Affymetrix PrimeView chips. As shown with the volcano plot in Figure 3a, there were 143 jointly downregulated genes (blue point) and 154 commonly upregulated genes (red points) in CDDP-pretreated A549, NCI-H460 and NCI-H1299 cells. Interestingly, we found some upregulated genes were related to promote tumor proliferation and migration, suggesting CDDP-induced enrichment of CSCs might be caused by gene alteration (Supplementary Figure 2A). Additionally, genechip data was confirmed by quantitative real-time PCR (Supplementary Figure 2B). To further explore the possible pathway involved in the CDDP-induced enrichment of CSCs, gene set enrichment analysis (GSEA) was used to compare the DMSO treated with CDDP-treated A549, NCI-H460 and NCI-H1299 cells. This analysis revealed an enrichment of signatures comprising genes involved in MAPK pathway in CDDP-pretreated cells (Figure 3b). Among those MAPK pathway related genes, TRIB1, a pseudokinase, was found obviously overexpressed in CDDP-pretreated cell lines (Figure 3c). For validation we analyzed the expression level of TRIB1 in CDDP-treated and -untreated NSCLC cell lines and confirmed the upregulation of TRIB1 at both mRNA and protein levels in CDDP-pretreated three NSCLC cell lines (Figure 3d; Supplementary Figure 2B). A crucial role of TRIB1 in MAPK pathway is conducted to promote ERK phosphorylation,^20^ so we also detected the phosphorylated ERK in CDDP-treated and -untreated cells. As shown in Figure 3d and Supplementary Figure 3, the level of phosphorylated ERK was markedly increased in CDDP-treated NSCLC cells. The above data indicate TRIB1 is overexpressed in CDDP-treated NSCLC cells.

To test whether TRIB1 is indeed involved in the observed CDDP-induced CSCs enrichment and resistance, we knockdown TRIB1 using specific siRNA. As shown in Figures 3e–g, silence of TRIB1 resulted in a robust downregulation of CSCs transcription factors, a significant reduction of CDDP-induced sphere formation, as well as an enhanced sensitivity to CDDP itself in NSCLC cells. Thus, our results suggest that TRIB1 contribute to CDDP-induced CSCs enrichment and resistance in NSCLC cells.

C/EBP-*β*, as a transcription factor, was reported to positively regulate the expression level of TRIB1.^21^ To address whether the overexpression of TRIB1 is related to C/EBP-*β*, we first detected the expression level of C/EBP-*β* in CDDP-treated and -untreated NSCLC cell lines. Western blot data revealed that pretreatment with CDDP could induce the expression of C/EBP-*β* in three NSCLC cell lines (Figure 3d; Supplementary Figure 3). Additionally, chromatin immunoprecipitation (ChIP) data demonstrated, among four candidate binding site of TRIB1 gene promoter and enhancer (P1 to P4), pretreated with CDDP contributed to a substantial enrichment of C/EBP-*β* in P1 and P4 in NCI-H460 cells (Figure 3h), suggesting the potential role of C/EBP-*β* in TRIB1 transcriptional regulation. Consistent with these findings, the silence of C/EBP-*β* by specific siRNA resulted in a reduction of TRIB1 in CDDP-pretreated A549 and NCI-H460 cell lines (Figure 3i). Taken together, the results demonstrate that overexpression of TRIB1 in CDDP-pretreated NSCLC cells is dependent on C/EBP-*β* regulation.

### HDAC cooperated with TRIB1 involved in CDDP-induced CSCs enrichment and resistance in NSCLC cells

**Figure 4:**
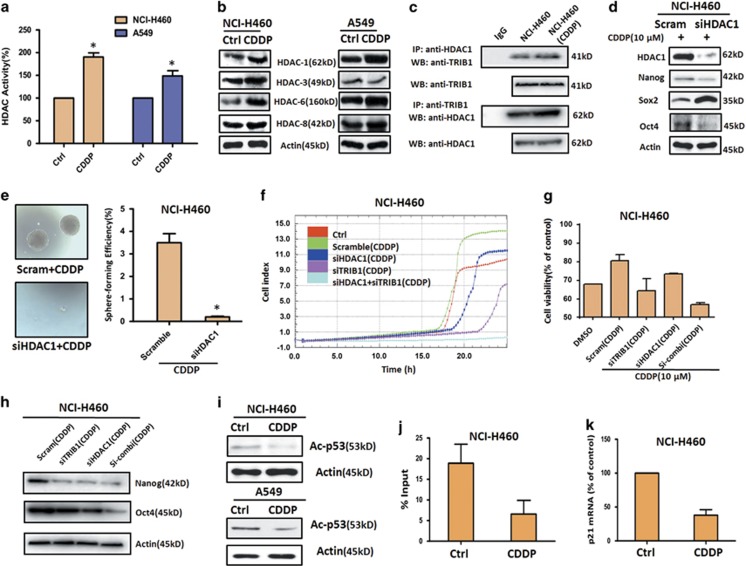
HDAC activity and role in CDDP-induced CSCs enrichment and resistance in NSCLC cells. (**a**) The HDAC activity in CDDP-pretreated and DMSO-pretreated NSCLC cell lines. (**b**) HDAC1, HDAC3, HDAC6 and HDAC8 were measured in CDDP-pretreated and DMSO-pretreated NSCLC cell lines. *β*-Actin expression was used as a loading control. (**c**) The interaction between HDAC1 and TRIB1 was estimated using co-immunoprecipitation assays in NCI-H460 cells. The expression CSCs transcription factors (**d**) and sphere formation (**e**) were accessed in CDDP-pretreated and DMSO-pretreated NCI-H460 cells after knockdown HDAC1 by siRNA (50 nM). The migration (**f**), sensitivity to CDDP (**g**) and expressions of CSCs transcription factors (**h**) were accessed in CDDP-pretreated and DMSO-pretreated NCI-H460 cells after single knockdown HDAC1, TRIB1 or combined silence by siRNA (HDAC:50 nM, TRIB1:33 nM). (**i**) The expression level of Ac-p53 was detected by western blot in CDDP-pretreated and control NCI-H460 and A549 cells. *β*-Actin expression was used as a loading control. (**j**) ChIP assays revealed the difference of p53 binding ability to the promoter of p21 gene. (**k**) The mRNA of p21 was detected by real-time RT-PCR in CDDP-pretreated and control NCI-H460 cells. *β*-Actin expression was used as a control. All error bars are s.e.m. *P<0.05, compare with control

To understand how TRIB1-mediated CSCs enrichment and resistance, we next detected the role of HDAC, which is known as TRIB1 partner and also associated with drug resistance.^15, 22^ As shown in Figure 4a, pretreatment with CDDP resulted in an increase of HDAC activity in both NCI-H460 and A549 cells, with about twofolds and 1.5 folds increasing as compared with DMSO control. Consistent with the increase of HDAC activity, the subtypes of HDAC, including HDAC1, HDAC3, HDAC6 and HDAC8, were upregulated by CDDP pretreatment, especially in NCI-H460 cells (Figure 4b; Supplementary Figure 4A). Among the subtypes of HDAC, HDAC1 is considered as a dominant functional subtype and also a TRIB1-interacted protein. Thus, we next explore whether CDDP pretreatment could induce the enhanced interaction of TRIB1 with HDAC1. As shown in Figure 4c, the interaction of TRIB1 with HDAC1 was reinforced by CDDP pretreatment. The role of HDAC1 in CDDP-induced enrichment of CSCs and drug resistance was further detected after knockdown HDAC1. Western blot data showed that silence of HDAC1 contributed to a remarked reduction of Nanog and Oct4, but led to an upregulation of Sox2 (Figure 4d). Furthermore, silence of HDAC1 also led to an obvious reduction of CDDP-induced sphere formation and cell migration, and mediated an enhanced sensitivity to CDDP and NVB in NSCLC cells (Figures 4e and f; Supplementary Figures 4B and C). More importantly, the combined silence of HDAC1 and TRIB1 could completely block CDDP-induced cell migration, improve sensitivity to CDDP, and reduce the expressions of CSCs transcription factors, especially for Oct4, in NCI-H460 cells (Figures 4f–h), indicating the possibility that HDAC1 and TRIB1 cooperatively mediate CDDP-induced CSCs enrichment and resistance in NSCLC cells.

To further explore the cooperation mechanisms, we detected the acetylated expression and binding ability of p53 which is recently known as inhibitor of CSCs and could be regulated by TRIB1 and HDAC1.^23, 24, 25^ Our results revealed that pretreatment with CDDP led to a downregulation of acetylated p53 in both NCI-H460 and A549 cells (Figure 4i), which is consistent with the activation of TRIB1 and HDAC. Additionally, ChIP assay and real-time PCR data indicated that CDDP-pretreated NCI-H460 cells exhibited a reduced binding ability of p53 to p21 promoter, and displayed decreasing expression of p21 at mRNA level (Figures 4j and k), which further confirmed the activity of p53 was downregulated by pretreatment with CDDP in NSCLCs.

### HDAC inhibitor combined with CDDP synergistically suppressed tumor growth of NSCLCs *in vitro* and *in vivo*

**Figure 5:**
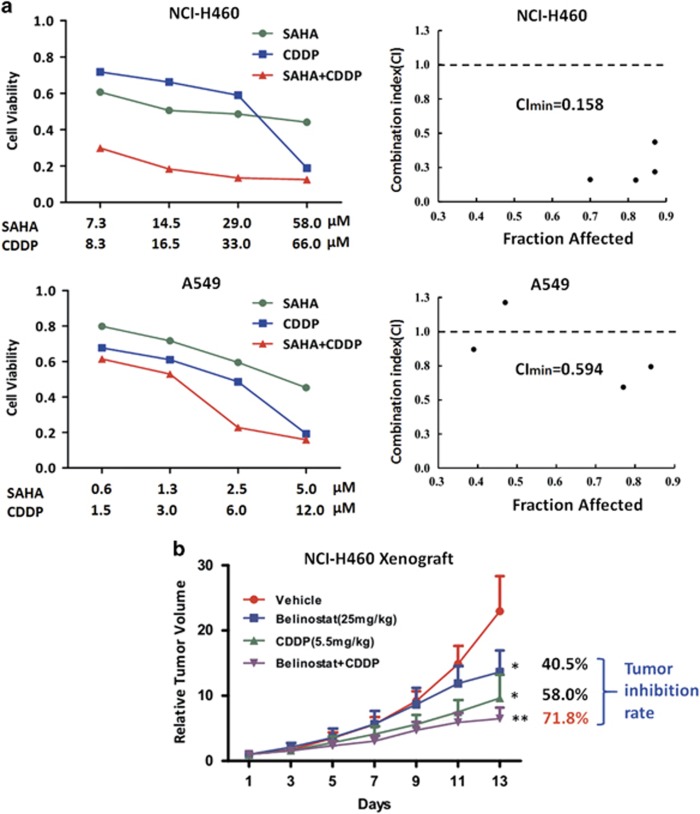
The effects of HDAC inhibitor, CDDP and their combination on the growth NSCLC cells *in vitro* and *in vivo*. (**a**) The growth curve of NCI-H460 cells and A549 cells after treated with SAHA, CDDP and the combination of SAHA and CDDP. Analysis of the combination of SAHA and CDDP in both cell lines. The cells were treated for 48 h using increasing concentrations of SAHA and CDDP, either alone or in a fixed ratio. The resultant data were analyzed using Calcusyn program, and graphs from the averaged results of three independent experiments are shown. (**b**) The relative tumor volume of NCI-H460 xenografts after administrated with belinostat (25 mg/kg/2days, i.p.), CDDP (5.5 mg/kg/3-4days, i.p.) and the combination of belinostat and CDDP. *P<0.05, **P<0.01, compare with control

Because HDAC activity and expression were increased in CDDP-pretreated NSCLC cells, we next asked whether ablation of HDAC could sensitize NSCLC cells to CDDP *in vitro* and *in vivo*. MTT data showed the combination of SAHA, a HDAC inhibitor and CDDP could obviously inhibit cell growth as compared with SAHA or CDDP alone (Figure 5a). The drug combination effect analysis that revealed synergistic effects occurred between SAHA and CDDP. For instance, the minimal combination index (CI) value for the SAHA-CDDP was 0.158 in NCI-H460 cells and 0.594 in A549 cells, respectively (Figure 5a). To address the role of TRIB1 in CDDP resistance, we further detected the combination effect after knockdown TRIB1. As shown in Supplementary Figures 5A and B, silence of TRIB1 by siRNA could partially reverse the synergistic effect of the combination SAHA and CDDP in NCI-H460 and A549 cells. Next, we evaluated the antitumor effect of this combination strategy *in vivo*. After tumors reached an average volume of 80 mm^3^, the BALB/c mice bearing NCI-H460 xenografts were treated with CDDP alone, HDAC inhibitor (Belinostat) alone, or dual combinations (Belinostat plus CDDP). Xenografts treated with Belinostat showed a weak inhibition, with the tumor inhibition rate 40.5% (Figure 5b). Additionally, CDDP administration could result in a moderate suppression of tumor growth during the course of the experiment, with the tumor inhibition rate 58.0% (Figure 5b). Notably, mice treated with the dual combination exhibited a 71.8% inhibition, therefore mirroring our *in vitro* results (Figure 5b). These results indicate that the ablation of HDAC could sensitize NSCLC cells to CDDP *in vitro* and *in vivo*.

### Overexpression of TRIB1 associated with unfavorable response to CDDP-based chemotherapy in NSCLC patients

**Figure 6:**
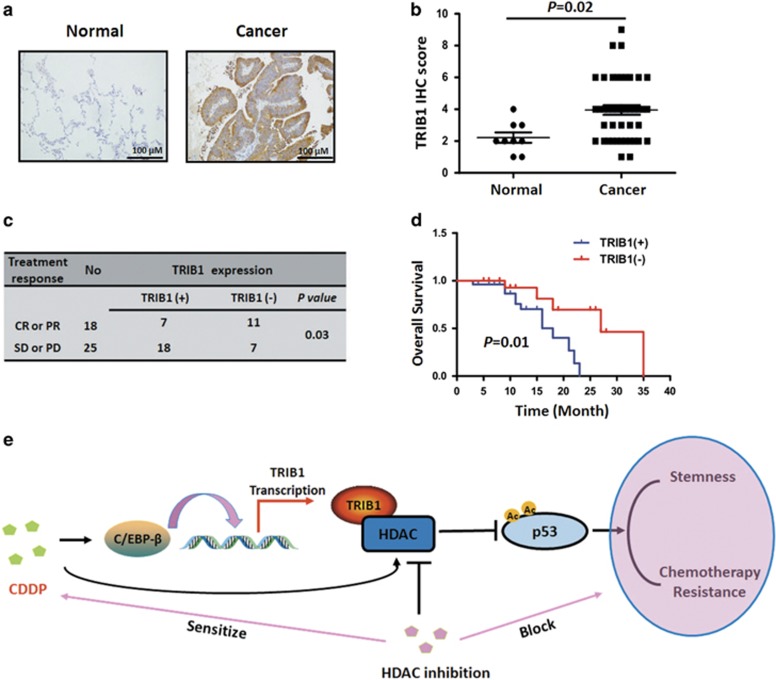
TRIB1 expression in human normal lung tissues and NSCLCs treated with CDDP. (**a**) The expression of TRIB1 in representative NSCLC tissues and adjacent normal tissues. Figures magnified 200 × . (**b**) The difference of expression level of TRIB1 in NSCLC tissues and adjacent normal tissues. (**c**) The correlation between treatment response and the expression of TRIB1 in NSCLC. CR, complete response; PR, partial response; SD, stable disease; PD, progressive disease (**d**) Overall survival according to expression of TRIB1 in NSCLCs. (**e**) Schematic figure of CDDP enriching CSCs and inducing chemotherapy resistance in NSCLCs

To understand whether TRIB1 expression could be related to CDDP-based chemotherapy, immunohistochemistry was performed in 43 lung tumors from NSCLC patients with CDDP-based chemotherapy and 9 paired normal tissue. TRIB1 protein expression was elevated in the NSCLC samples compared with the adjacent normal tissues (Figures 6a and b). Additionally, cBioPortal database analysis further revealed that frequency of the *TRIB1* gene amplification in lung cancer specimens was 6 to 7% (Supplementary Figure 6A), suggesting a prooncogenic role of TRIB1 in NSCLC. Interestingly, our data also indicated that the higher expression of TRIB1 was correlated with bad treatment response and poor overall survival than lower expression of TRIB1 group in lung cancer (Figures 6c and d). Furthermore, cBioPortal database analysis also showed that amplification of *TRIB1* was marginally related to a bad disease free survival of lung cancer patients (Supplementary Figure 6B, *P*=0.059). These results are consistent with the above findings, and suggest that TRIB1 may serve as a predictive marker for CDDP efficacy and their changes may be associated with CDDP resistance.

## Discussion

In the present study, we found that CDDP, but not Taxel and DOX, enriched CSC of NSCLC *in vitro* and *in vivo*. Mechanistically, CDDP pretreatment resulted in upregulation of TRIB1 through activating transcription factor C/EBP-*β*. Additionally, CDDP pretreatment also contributed to activation of HDAC, which is known as TRIB1-interacted protein. The crucial role of TRIB1 and HDAC in CDDP-induced enrichment of CSC and resistance was verified by knockdown TRIB1, HDAC1, and the combined silence. Notablely, CDDP-treated NSCLC patients with high levels of TRIB1 showed a significantly bad response and poorer prognosis, which further confirmed the role of TRIB1 in CDDP resistance. Importantly, the combination of HDAC inhibitor and CDDP displayed a reinforced antitumor action in NSCLC cell lines and xenograft models. Our findings illustrate a novel perspective in the evolution of CDDP resistance and provide a promising approach for the treatment of patients with NSCLC.

Platinum-based chemotherapy for advanced NSCLC is considered as the first line option,^26^ but in neo-adjuvant chemotherapy trials pathological complete RRs are ~10%.^27^ This indicates either *de novo* or acquired resistance to platinum and shows a mainly barrier to improving long-term outcomes. Multiple mechanisms seem to be at play in platinum resistance, such as the loss copper transporter CTR1, which results in the less platinum entering the cells,^28^ upregulation of ERCC1 that remedies platinum-DNA damage,^29^ and activation of survival pathway.^26^ However, there was still only limited evidence about the cell phenotypic mechanism. Here, mimic clinical administration dose, pretreatment NSCLC cells with CDDP under plasma concentration for a relative longer time, resulted in an increase of colony number and sphere formation, contributed to an enhanced expression or activity of CSCs' transcription factors and CSCs' biomarkers, and led to resistance to chemotherapy agents, suggesting CDDP pretreatment could enrich CSCs in NSCLC. In consistent with our results, Zhang *et al.*^30^ recently reported that CDDP treatment could increase sphere formation and CSC markers in NSCLC cells. Interestingly, our findings also showed that CDDP pretreatment conferred to the reinforced migratory capability of NSCLC cells, which, to some extent, explains the enhancement of distant relapse after CDDP administration in clinical study. In contrast to CDDP, Taxel and Dox pretreatment could not result in an obvious enrichment of CSC in NSCLC. The possible reason might be explained by the following facts: (1) according to action mechanism, CDDP-induced DNA cross-links are primarily bypassed during replication through translesion DNA synthesis (TLS),^31^ which in turn results in the enrichment of CSC;^32^ (2) compared with CDDP, Dox and Taxol might need a relative long time or a low concentration to trigger CSCs phenotype. Previous studies show Dox or Taxol with long time exposure or low concentration treatment exhibits CSCs phenotype.^33, 34^ In line with these reports, our *in vivo* data also showed three weeks administration of Dox led to upregulation of CSCs transcription factors. However, the underlying reason need further be investigated.

TRIB1 has been identified as oncogene that mediates oncogenesis through the MEK–ERK pathway, GRP78–Akt pathway and C/EBP regulation.^12, 13, 14^ In the present study, our GSEA analysis data demonstrated that TRIB1, as MAPK pathway related gene, was upregulated by CDDP pretreatment in NSCLC cells. We showed that siRNA-mediated TRIB1 knockdown led to the reverse of CDDP-enriched CSC phenotype, including sphere formation ability, expression levels of CSCs' markers, and resistant capacity to chemotherapy agent. These results supported TRIB1 as an induced target gene important in regulating CDDP-enriched CSC and drug resistance. In addition, the expression level of TRIB1 in NSCLC tissues was higher than normal lung tissues (*P*<0.05), and corrected with treatment response and prognosis of CDDP administrated NSCLC patients. Furthermore, our data are consistent with the findings based on cBioPortal database, which showed that the amplification of *TRIB1* gene was associated with disease free survival, implicating *TRIB1* may function as an oncogene involved into CSC phenotype maintenance and chemotherapy resistance.

HDAC is increasingly recognized as a major factor contributing to pathogenesis of cancer including NSCLC.^35^ Recently, it was reported that the HDAC1, a dominant subtype of HDAC, interacts with TRIB1 to suppress p53 transcriptional activity by enhancing deacetylation and decreasing DNA binding.^25^ Meanwhile, our previous results indicate that HDAC1 is involved into paclitaxel resistance in NSCLC.^22^ Therefore, we hypothesis that TRIB1 cooperated with HDAC1 mediate CDDP-enriched CSC. Here, our results revealed that either activity or expression level of HDAC was increased in CDDP-pretreated NSCLC cells. The interaction of HDAC1 with TRIB1 was also enhanced after CDDP pretreatment. Furthermore, acetylated p53 and its binding ability were reduced by CDDP pretreatment. Additionally, silencing of HDAC resulted in reduction of CDDP-induced CSC, and combined knockdown of HDAC1 and TRIB1 exhibited enhanced effect. Overall, these results verified our hypothesis that TRIB1 cooperated with HDAC1 regulate p53 activation, subsequently mediate CSC enrichment and chemotherapy resistance induced by CDDP.

The frequent resistance to CDDP in NSCLC has led to the development of novel therapeutic strategies. The increase of HDAC activity and expression in CDDP-pretreated NSCLC cells prompted us to evaluate the combination strategy *in vitro* and *in vivo*. Our data indicated that HDAC inhibitor in combination with CDDP exerted promising activity to overcome tumor growth *in vitro* and in preclinical models. These data is consistent with previous study in clinic, which showed 100% partial response to HDAC inhibitor combined with CDDP-based chemotherapy in patients with inoperable stage III NSCLC.^36^ Mechanistically, the elimination of cancer stem cell by regulating HDAC and TRIB1 might be a reason. However, considering the minor ratio of cancer stem cell in total tumor cells, other mechanisms might have the crucial role in this process. In fact, several reports has been shown that SAHA and other HDAC-inhibitors enhances tumor cell apoptosis through induction of proapoptotic proteins, such as Bim, Noxa and PUMA.^37, 38^ Therefore, it could not denied the possibility that induction of apoptosis, but not suppression of cancer stem cell, by HDAC inhibitor is mainly involved in these effects.

In conclusion, the present study discloses that the chemotherapy agents, especially CDDP, themselves could enrich CSC, whereby result in the enhancement of malignant phenotype. The CDDP-enriched CSC is associated with an aberrant activation of the C/EBP-*β*/TRIB1/HDAC/p53 axis (Figure 6e). Moreover, the combination therapy of HDAC inhibitor and CDDP shows a reinforced effect both *in vitro* and *in vivo*. Our data not only elucidated an additional mechanism for tumor recurrence and acquired resistance in CDDP-treated NSCLC but also provided a promising therapeutic strategy to reduce the recurrence of NSCLCs.

## Materials and Methods

### Cell lines, cell culture and treatment

Human lung cancer cell lines NCI-H460, A549 and NCI-H1299 were obtained from the American Type Culture Collection (Manassas, VA, USA). These cancer cells were routinely cultured in RPMI-1640 or MEM medium supplemented with 10% fetal bovine serum and maintained at 37 °C in a humidified incubator with 5% CO_2_. The cells were treated chemotherapy agents for 72 h, and then recovered for 48 h, and next prepared for the biological study.

### Patients and chemotherapy

A total of 43 patients with advanced NSCLC (stage IIIB and stage IV) were enrolled between January 2004 and June 2012 from Wuhan General Hospital of Guangzhou Command (Wuhan, China), and the detailed information was shown in Supplementary Materials and Methods. Ethical oversight and approval was obtained from the Institutional Review Board of Wuhan General Hospital of Guangzhou Command.

### Immunohistochemistry

A tissue microarray was constructed (in collaboration with the Shanghai Biochip Company Ltd.) as described previously.^22^ The paraffin sections were dewaxed with xylene and rehydrated in descending concentrations of ethanol. The endogenous peroxidase was inhibited, and the slides were incubated with antibody against TRIB1 (1:200; Abcam, Cambridge, UK). The expression level of TRIB1 was graded as described in Wang *et al.*^39^

### Compounds and reagents

Cisplatin (CDDP), paclitaxel (Taxel), doxorubicin (Dox), Vinorelbine (NVB) and SAHA were obtained from Sigma (St. Louis, MO, USA). Belinostat was synthesized in Medicine Chemistry Laboratory at Shenyang Pharmaceutical University (purity >97%). The primary antibodies against HDAC1, HDAC3, HDAC6, Nanog, Oct4, Sox2, ERK, phosphor-ERK and *β*-actin were got from Cell Signaling Technology (Beverly, MA, USA). The primary antibodies against CD133 was obtained from Mitenyi Biotec. The primary antibodies against TRIB1, C/EBP-*β*, HDAC8 and Acetyl-p53 were purchased from Abcam (Cambridge, MA, USA). Antibodies to ALDH1A1, ALDH1A3, ALDH2, ALDH3A1, ALDH5A1 and ALDH7A1 were obtained from Novus Biologicals (Littleton, CO, USA). The Silencer Select Validated siRNA, including TRIB1, HDAC1 and C/EBP-*β*, were got from Life technologies (Gaithersburg, MD, USA).

### Clonogenicity assay

The cells were treated with different chemotherapy agents. Then the cells were incubated for an additional 7 days. The colonies obtained were formalin fixed and stained with hematoxylin. The colonies were counted and compared with untreated cells.

### Tumorsphere formation assay

Single cells prepared from mechanical and enzymatic dissociation were seeded in 6-well ultra-low attachment plates (Corning, NY, USA) at 3000 cells per well for about 2 weeks containing serum-free DMEM/F-12 medium, B27 supplement (1 × , Invitrogen, Gaithersburg, MD, USA), 20 ng/ml human recombinant bFGF (PeproTech, Rocky Hill, NJ, USA), 20 ng/ml EGF (PeproTech), 10 ng/ml leukemia inhibitory factor (Chemicon, Tamecula, CA, USA) and 4 U/l insulin (Sigma).

### Western blot analysis

About 1–5 × 10^6^ cells were gathered after pretreatment for the indicated time periods as described previously. The detailed process was shown in Supplementary Materials and Methods.

### Real-time cell analysis

Real-time cell analysis assay was used to detect cell migration. The detailed protocol was shown in Supplementary Materials and Methods.

### Cell viability assay and determination of combination index

The *in vitro* cell viability was determined by MTT assay. The detailed process was shown in Supplementary Materials and Methods. The combination index was analyzed by Calcusyn software (Biosoft, Oxford, UK).

### Flow cytometry analysis

To examine expression of putative tumor-initiating cell markers, including ALDH and CD133, single-cell suspensions from CDDP-pretreated cells were isolated as outlined. The detailed protocol was shown in Supplementary Materials and Methods.

### RNA sequencing and gene expression analysis

Total RNA of CDDP-pretreated cells was isolated by using RNeasy Mini Kit (Qiagen, Valencia, CA, USA) as described in the product insert. Array hybridization was performed according to Affymetrix FS450_0002 Hybridization Protocol for gene expression. The Affymetrix GeneChip PrimeView Human Gene Expression Arrays were scanned with Affymetrix Genechip Scanner 7G. For quantitative PCR (qPCR), the RNA was reverse transcribed by using random hexamer primers and a revertAid first-strand cDNA synthesis kit (Invitrogen). qPCR was performed by using iTaq Universal SYBR Green Supermix (Bio-Rad, Hercules, CA, USA). The sequences of qPCR primers are listed in Supplementary Table 1.

### HDAC whole-cell assay

The cells were treated as described above before assays. The HDAC activity was measured with a HDAC fluorescent activity assay kit (Biovison Mountain View, CA, USA) according to the recommended manual.

### ChIP assay

Using ChIP Assay Kit (Beyontime, CHN), NCI-H460 cells were prepared for the ChIP assay performed as the instructions of the manufacturer. The detailed protocol was shown in Supplementary Materials and Methods.

### Co-immunoprecipitation

NCI-H460 cells were treated with DMSO or CDDP as described above, and then harvested. Next, cells were lysed with lysis buffer and immunoprecipitated with the indicated antibodies. Proteins were resolved by SDS-PAGE gels and detected by western blot. Equal amounts of samples were mixed with normal IgG as negative controls.

### Mouse xenograft tumors study

NCI-H460 xenograft models were established and used to assess characteristics assessment of chemotherapy resistant tumors and combined treatment efficacy *in vivo*. The detailed experiments were described in Supplementary Materials and Methods. These studies were performed in strict accordance with the recommendations in the Guide for the Care and Use of Laboratory Animals of the National Institutes of Health. The protocol was approved by the Committee on the Ethics of Animal Experiments of the Shenyang Pharmaceutical University.

### Statistical analysis

Differences between experimental groups were evaluated by one-way ANOVA or Turkey's *post hoc* test using the SPSS11.5 software package for Windows (SPSS, Chicago, IL, USA). Survival curves were constructed using the Kaplan–Meier method. Statistical significance was based on a *P*-value of 0.05 (*P*<0.05, two-tailed test).
