# Supplementary material for: Cisplatin-enriching cancer stem cells confer multidrug resistance in non-small cell lung cancer via enhancing TRIB1/HDAC activity
Source: Cell Death Dis. 2017 Apr 13;8(4):e2746–. doi: 10.1038/cddis.2016.409 (PMC5477570; doi:10.1038/cddis.2016.409)
Supplement: Supplementary Information [file cddis2016409x1.docx]

**Materials and Methods**

**Patients and chemotherapy**

A total of 43 patients with advanced NSCLC(stage IIIB and stage IV) were enrolled between January 2004 and June 2012 from Wuhan General Hospital of Guangzhou Command (Wuhan, PR, China). The enrolled patients met the following eligibility criteria: histological or cytological confirmation of NSCLC, presence of measurable disease, no adjuvant or neoadjuvant therapy and surgery, no second malignancies, availability of adequate diagnostic tumor tissue (taken from brochoscopic biopsy, percutaneous lung biopsy or metastatic sites). Among 43 patients, included 29 male and 14 female, 7 squamous carcinoma cases and 36 adenocarcinoma cases, and the mean age was 66 years old.

Each patient underwent the treatment with at least two cycles of the first-line cisplatin-based chemotherapy. Response to treatment was determined after 2-3 cycles by RECIST (Response Evaluation Criteria in Solid Tumors) criteria, which classified the responses into complete response (CR), partial response (PR), stable disease (SD), and progressive disease (PD). SD plus PD were considered as chemotherapy resistance. Overall survival (OS) was defined as the time between the onset of chemotherapy and the date of the last follow up or death from any cause. Ethical oversight and approval was obtained from the Institutional Review Board of Wuhan General Hospital of Guangzhou Command.

**Real-time cell analysis（RTCA）**

The CIM-plate contains 16 modified Boyden chambers, which can be used independently to measure cell migration in real time through 8 μm pores of a polyethylene terephthalate membrane onto gold electrodes on the underside of themembrane using the xCELLigence analyser system (ACEA Biosciences). Experiments were set up according to the manufacturer's instructions with the membrane uncoated (migration). A chemotactic signal for movement was provided by inoculating 30,000–50,000 NCI-H460 cells in serum-free medium in the upper chamber and supplying 10% FBS in the lower chamber (10 ng/ml VEGF with the relevant concentration of drug). Cell index (electrical impedance) was monitored every 5 min for the duration of the experiment. The cell index represents the capacity for cell migration, and the slope of the curve can be related to the migration velocity of tumor cells. The cell index thus reflects the tumor cell’s migratory capacity.

**Cell viability assay and Determination of combination index**

The *in vitro* cell viability was determined by MTT assay. The cells (8×10^4^ cells/ml) were seeded into 96-well culture plates. After overnight incubation, the cells were treated with various concentrations of agents for 24 or 48h. Then 10 μl MTT solution (2.5 mg/ml in PBS) was added to each well, and the plates were incubated for an additional 4 h at 37 ^o^C. After centrifugation (2500 rpm, 10 min), the medium with MTT was aspirated, followed by the addition of 100 μl DMSO. The optical density of each well was measured at 570 nm with a Biotek Synergy^TM^ HT Reader.

A549 and NCI-H460 cells were treated with different concentrations of single SAHA, CDDP, or their combination. The cell viability was measured by MTT assay. The nature of the drug interaction was analyzed by using the combination index (CI) according to the method of Chou and Talalay. A CI value lower than 0.90 indicates synergism; a CI value between 0.90 and 1.10 indicates additive; and a CI value higher than 1.10 indicates antagonism. Data analysis was performed by the Calcusyn software (Biosoft, Oxford, UK).

**Flow cytometry analysis**

Aldefluor assay was done according to the manufacturer's guidelines (Stem Cell Technologies). Single cells obtained from cell cultures were incubated in an Aldefluor assay buffer containing an ALDH substrate, bodipy-aminoacetaldehyde (1 μmol/L per 1,000,000 cells), for 30 to 60 min at 37°C. As a negative control, a fraction of cells from each sample was incubated under identical condition in the presence of the ALDH inhibitor diethylaminobenzaldehyde (DEAB). Flow cytometry was used to measure ALDH-positive cell population. Desired cell populations were isolated using a FACSAriaⅢ flow cytometer (BD Biosciences).

For CD133 measure, cultured cell lines were trypsinized and resuspended. Following incubation with FcR blocking reagent (Miltenyi Biotec), cells were stained with anti-CD133. After washing, cells were fixed by incubation in paraformaldehyde (4%) for 60 minutes and analyzed using a LSRII (BD Biosciences) within 24 hours. Data were analyzed using FACSAriaⅢ flow cytometer (BD Biosciences).

**Chromatin immuno-precipitation assay (ChIP)**

Using ChIP Assay Kit (Beyontime, CHN), NCI-H460 cells were prepared for the ChIP assay performed as the instructions of the manufacturer. C/EBPβ antibody or p53 antibody were used to immunoprecipitation, respectively. *TRIB1* and *p21^WAF-1^* promoter primers were used to carry out PCR from DNA isolated from ChIP experiment. The quantitative PCR was used to analyze amplification product. The sequences of qPCR primers are listed in Table S1.

**HDAC activity assay**

For cell-based HDAC activity assay, the A549 and NCI-H460 cells were pretreated with CDDP as above described before assays. Proteins were isolated by using cell lysis buffer (Beyontime, CHN). The protein concentration was measured by BCA protein assay (Beyontime, CHN). Nuclear proteins were incubated with HDAC fluorimetric substrate at 37°C for 30 min. The HDAC assay developer (which produces a fluorophore in reaction mixture) was added, and the fluorescence was measured using a microplate reader (Molecular Devices). HDAC activity is presented as the means±SEM of three determinants.

**Western blot analysis**

About 1-10×10^6^ cells were gathered after pre-treatment for the indicated time periods as described previously. Briefly, an equal amount of total protein extracts from cultured cells or tissues were fractionated by 10-15% SDS-PAGE and then electrically transferred onto polyvinylidene difluoride (PVDF) membranes. Mouse or rabbit primary antibodies and appropriate horseradish peroxidase (HRP)-conjugated secondary antibodies were used to detect the designated proteins. The bound secondary antibodies on the PVDF membrane were reacted with ECL detection reagents (Pierce; Rockford, USA) and exposed to X-ray films. Results were normalized to the internal control β-actin.

**Mouse xenograft tumors study**

For the characteristics assessment of chemotherapy resistant tumors, the viable NCI-H460 cells (5×10^6^/100 μl PBS per mouse), as confirmed by trypan blue staining, were subcutaneously injected into the right flank of 7- to 8- week old male BALB/c mice. When the average tumor volume reached 50 mm^3^, the mice were randomly divided into three treatment groups, including control (saline only, n=6), Dox (2.2 mg/kg/3-4days, i.v.; n=6), CDDP (5.5mg/kg/3-4days, i.v.; n=6). The dose of Dox and CDDP was calculated by clinical equivalent amount. After 15 days, the mice were sacrificed and the tumors were excised and stored at -80°C until western blotting.

To determine the *in vivo* anti-tumor activity of HDAC inhibitor, belinostat, combined with CDDP, viable NCI-H460 cells (5×10^6^/100 μl PBS per mouse) were subcutaneously injected into the right flank of 7- to 8- week old male BALB/c mice. When the average tumor volume reached 80 mm3, the mice were randomly divided into four treatment groups, including control (saline only, n=8), belinostat (25mg/kg/2days, i.p.; n=8), CDDP (5.5mg/kg/3-4days, i.p.; n=8), and the combination (n=8). Tumor size was measured once every three days with a caliper (calculated volume=shortest diameter^2^×longest diameter/2) for two weeks. These studies were performed in accordance with the recommendations in the Guide for the Care and Use of Laboratory Animals of the National Institutes of Health. The protocol was approved by the Committee on the Ethics of Animal Experiments of the Shenyang Pharmaceutical University.

Supple Tab.1 Realtime RT-PCR primer sequence

| Gene | Sequence | |  |
| --- | --- | --- | --- |
|  | Forward | Reverse |  |
| TRIB1 | TCTGCTGGATTTGGGAGTTC | TGTCATTGTGCGATTTGCTT | |
| ALDH3A1 | CTCTGTGACCCCTCGATCCA | GCATCTTCCCCGTAGAACTCTT | |
| CD68 | GGAAATGCCACGGTTCATCCA | TGGGGTTCAGTACAGAGATGC | |
| FGF1 | GAGCGAGTGTGGAGAGAGGT | GGCTGTGAAGGTGGTGATTT | |
| TAB3 | GCACTAAATCGCTGTGAGCA | TCCCGAGGTTTCCTTTTCTT | |
| β-actin | CTCCATCCTGGCCTCGCTGT | GCTGCTACCTTCACCGTTCC | |
| TRIB1_P1 | GGCCTGTGAGTGTGTGTGTA | GTGTATGAGAGCGAGCGAGA | |
| TRIB1_P2 | GCCAGGCTGGTCTCAAACT | GGACTTAGGCCAAGCACAGT | |
| TRIB1_P3 | ATCTGGGAGCTCTGAAGCTG | TTGCCTTGACTGAGAAGAGC | |
| TRIB1_P4 | CGCCTGTAATCCCAGCTACT | CGCCTAAGCTAGAGTGCAGTG | |
| p21_P1 | GTGGCTCTGATTGGCTTTCTG | CTGAAAACAGGCAGCCCAAG | |
| p21 | GTGGACCTGTCACTGTCTT | GCGTTTGGAGTGGTAGAAATC | |
