# Supplementary figures and images for: Cisplatin-enriching cancer stem cells confer multidrug resistance in non-small cell lung cancer via enhancing TRIB1/HDAC activity
Source: Cell Death Dis. 2017 Apr 13;8(4):e2746–. doi: 10.1038/cddis.2016.409 (PMC5477570; doi:10.1038/cddis.2016.409)

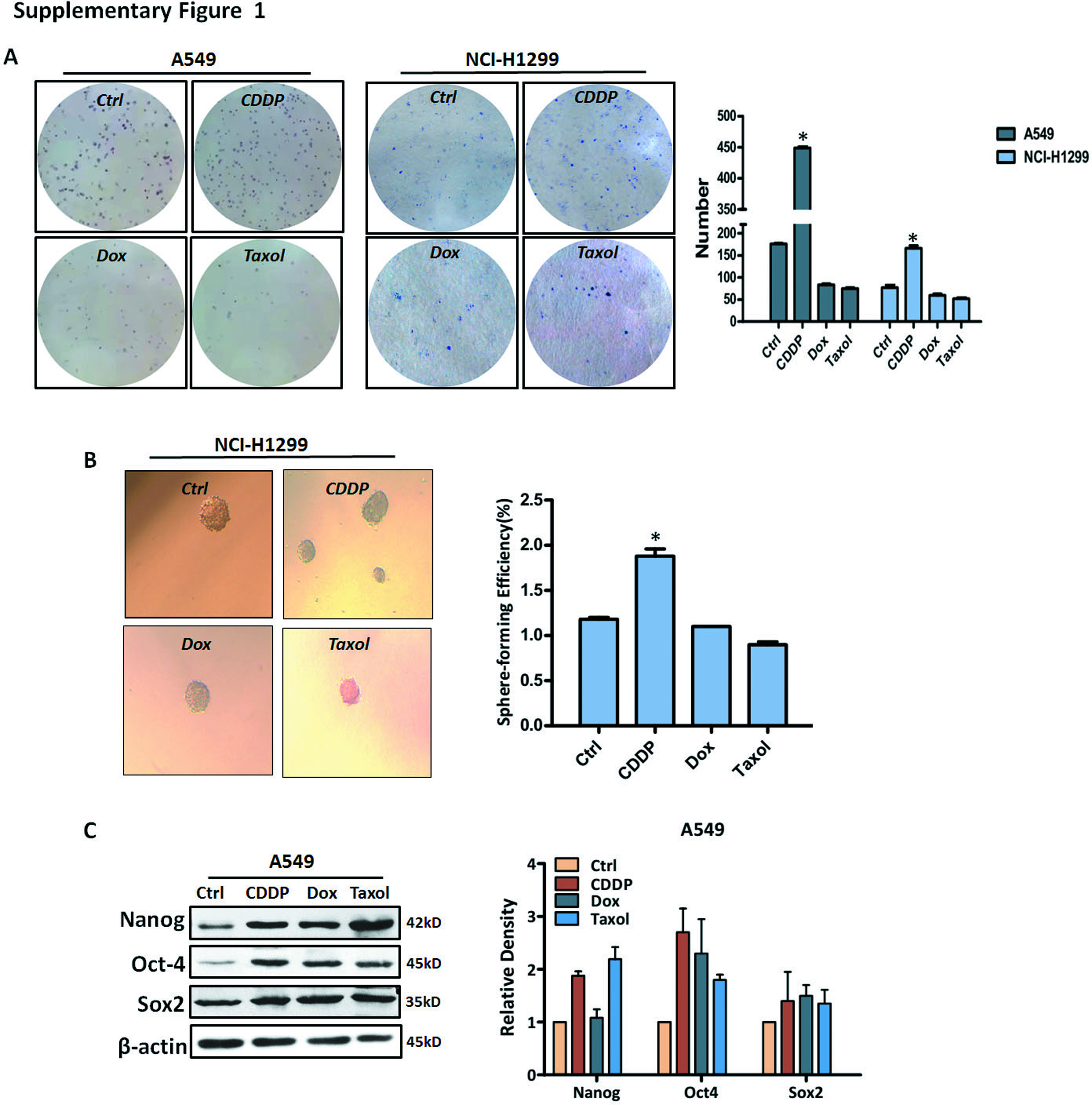

Supplement: Supplementary Figure 1 [file cddis2016409x2.tif]

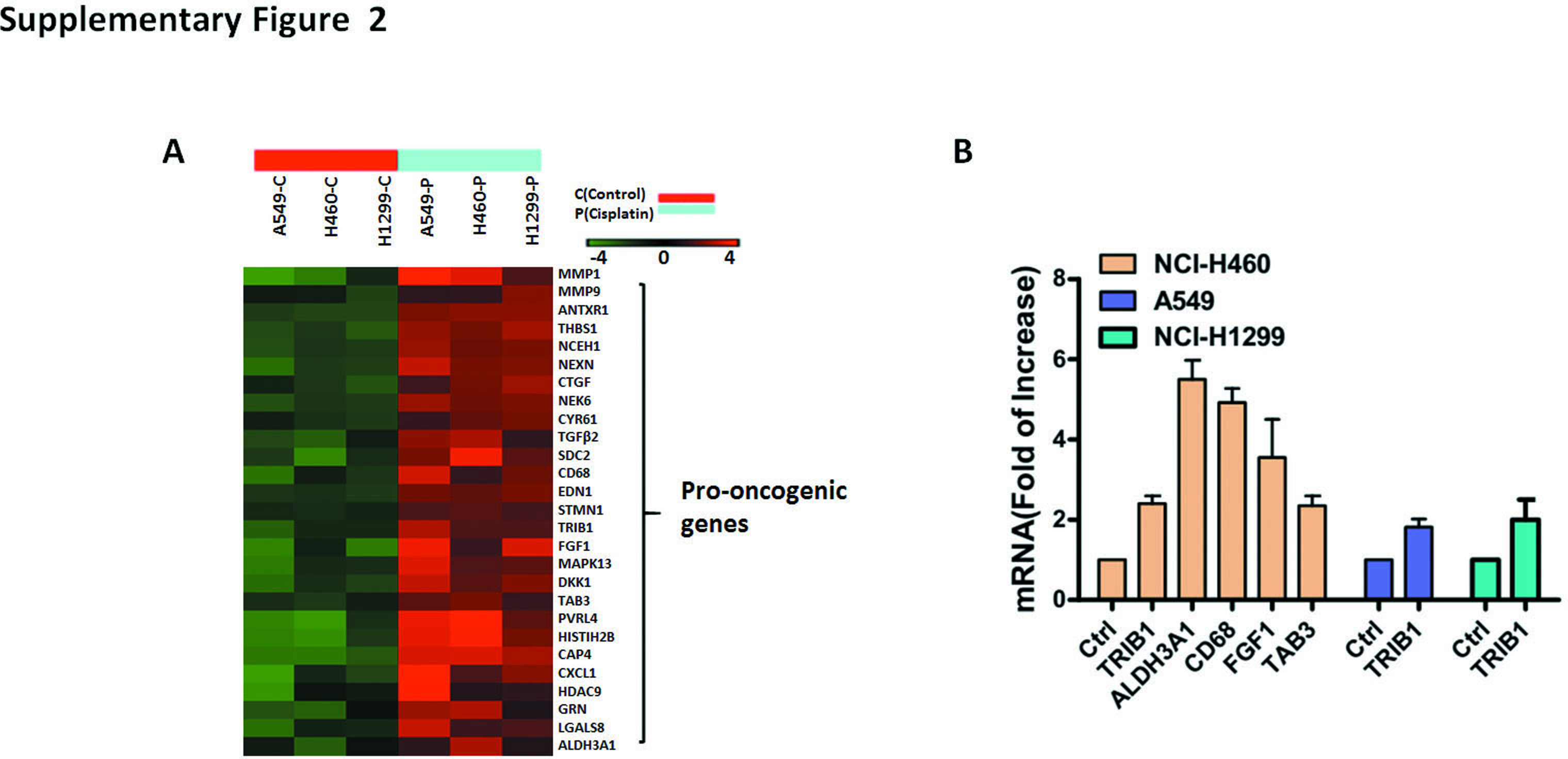

Supplement: Supplementary Figure 2 [file cddis2016409x3.tif]

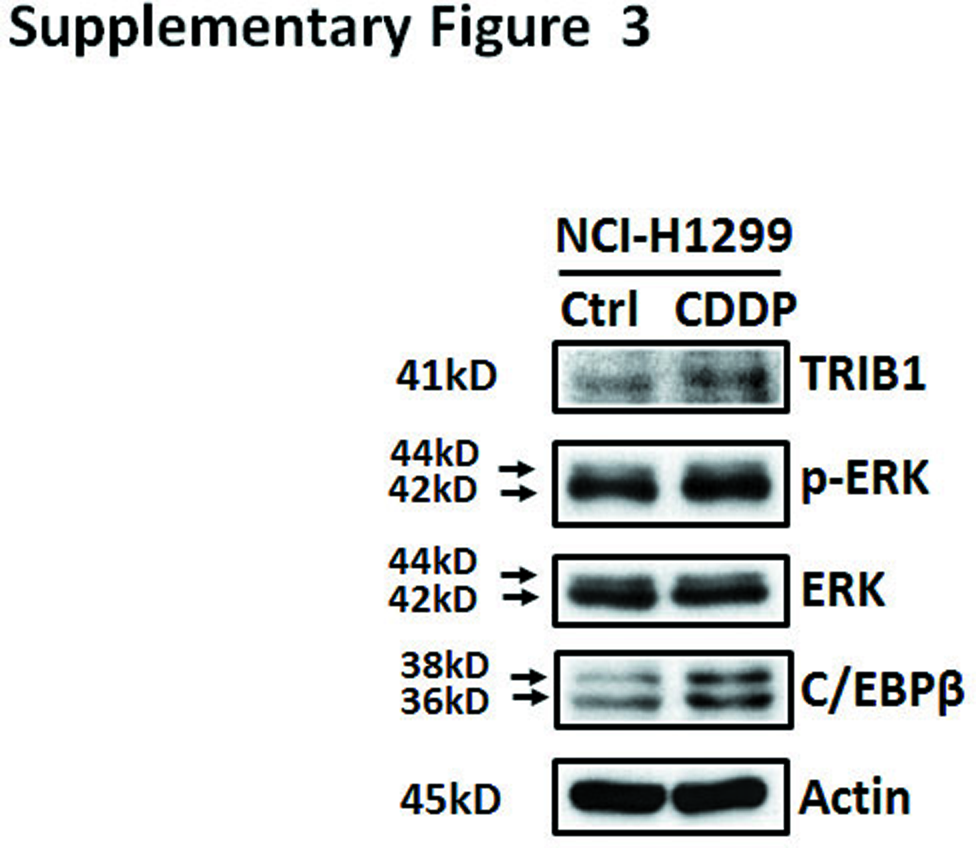

Supplement: Supplementary Figure 3 [file cddis2016409x4.tif]

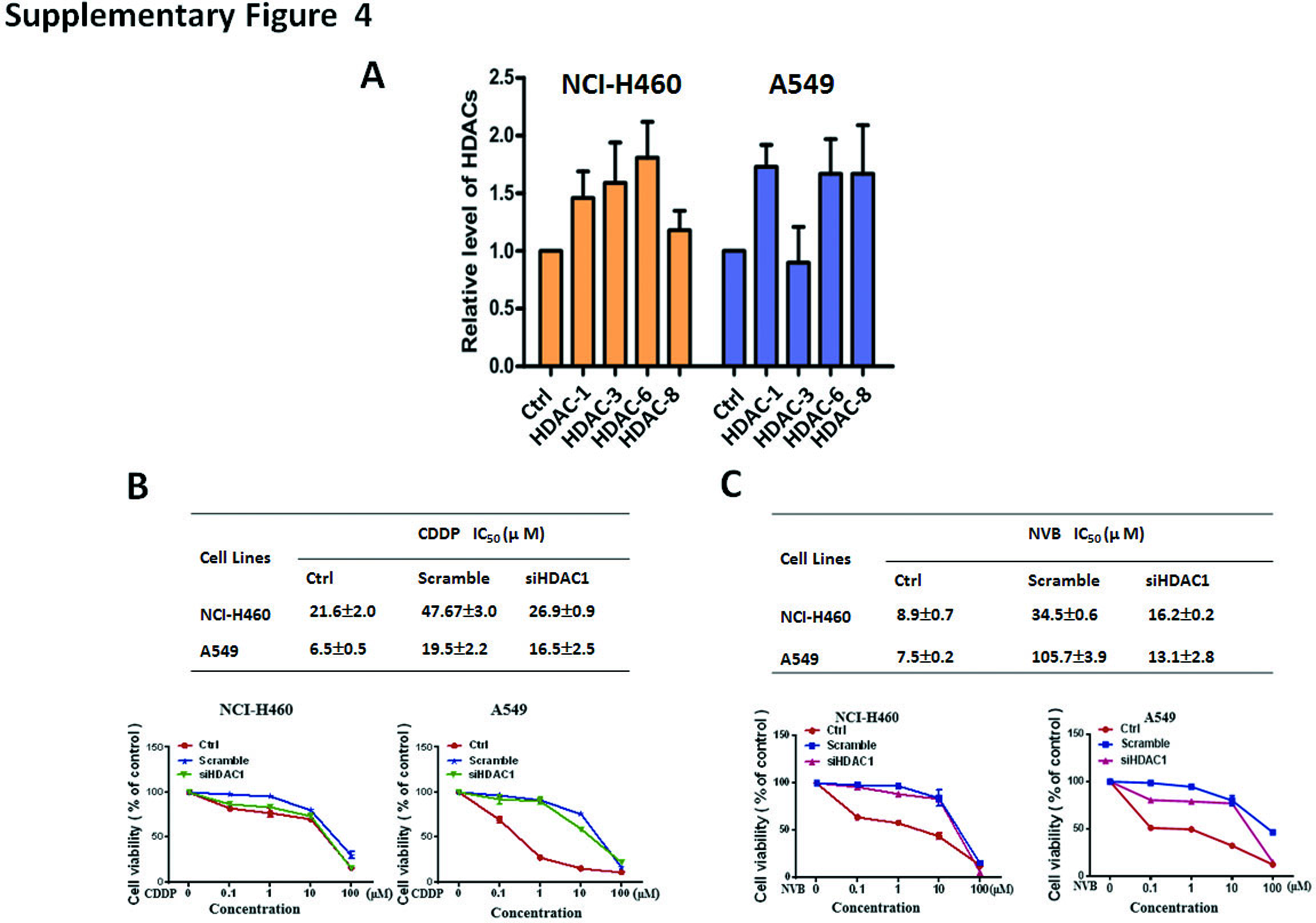

Supplement: Supplementary Figure 4 [file cddis2016409x5.tif]

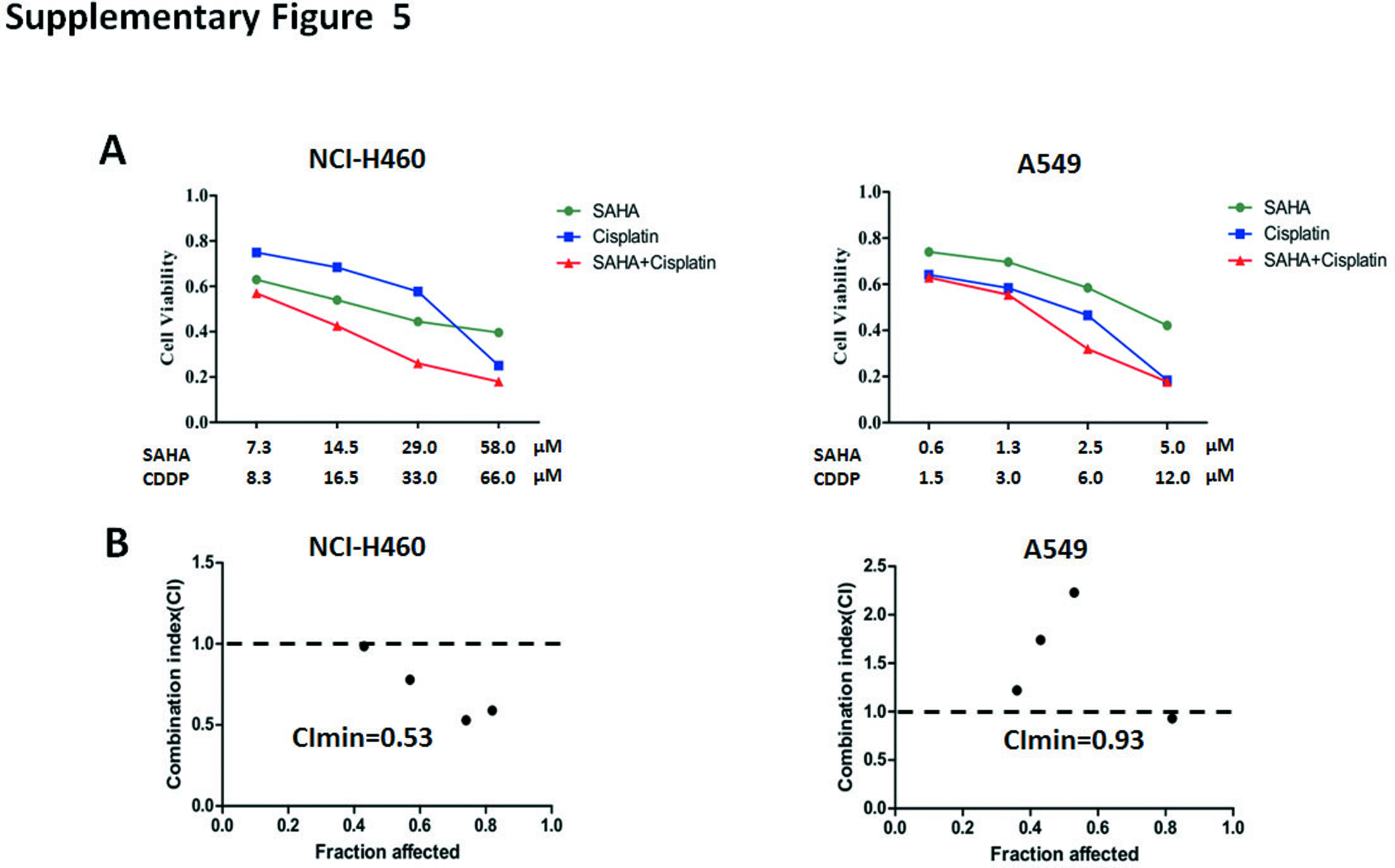

Supplement: Supplementary Figure 5 [file cddis2016409x6.tif]

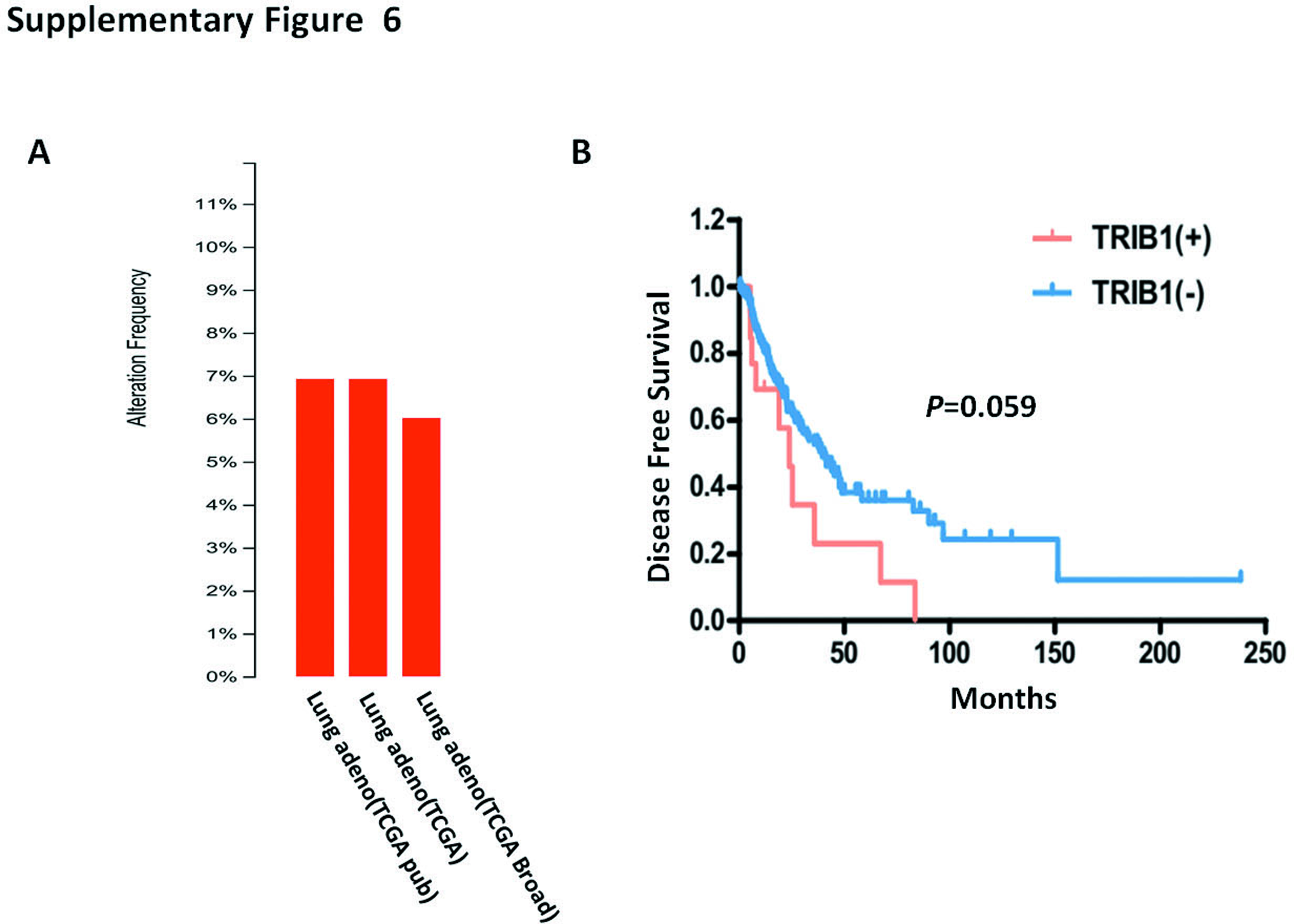

Supplement: Supplementary Figure 6 [file cddis2016409x7.tif]
